# Influence of faith-based organisations on HIV prevention strategies in Africa: a systematic review

**DOI:** 10.4314/ahs.v17i3.18

**Published:** 2017-09

**Authors:** Marylyn A Ochillo, Edwin van Teijlingen, Martin Hind

**Affiliations:** 1 Faculty of Health and Social Sciences, Bournemouth University; 2 Centre for Midwifery, Maternal & Perinatal Health, Faculty of Health & Social Sciences, Bournemouth University

**Keywords:** Faith-based organisations, HIV prevention strategies, systematic review

## Abstract

**Background:**

The HIV/AIDS epidemic remains of global significance and there is a need to target sub-Saharan Africa since it is the hardest hit region worldwide. Religion and more specifically faith-based organisations can have an effect on socio-cultural factors that increase or decrease the risk of infection; and offer preventative interventions to the wider community.

**Objective:**

To understand the influence of faith-based organisations on HIV prevention in Africa.

**Method:**

The main search engine of a British university ‘mysearch’ was used as this incorporates all relevant databases. Studies were also retrieved by searches within Google scholar, PubMed and reference lists of included papers were hand searched. The authors assessed the relevance of each article separately against the inclusion criteria. The data extraction form was piloted by the first author and cross-checked by the other authors.

**Results:**

Seven studies met all inclusion criteria and were reviewed. Seven individual themes were identified. However, for the purposes of focus within this paper only two themes were focused on.

**Conclusion:**

Given the accessibility of faith-based organisations (FBOs) and the coverage of religion among the population, FBOs are potentially important players in HIV prevention. Therefore, more resources and support should be given to support their health promotion strategies.

## Introduction

HIV (Human Immunodeficiency Virus) and AIDS (Acquired Immuno-Deficiency Syndrome) have some of the highest prevalence rates in sub-Saharan Africa according to the World Health Organization.^1^ Although the pandemic is slowing, more people are now living with HIV and this is partially explained by the increased survival rates due to improved access to anti-retroviral therapy (ART). The high numbers of people living with HIV/AIDS (PLWHA) is due to improved access to ART.^2^ Nevertheless, by virtue of ART's success rate, HIV has now become a chronic disease whereby the dominant problem is no longer that of dealing with its acute and life-threatening complications. Rather, clinicians are now confronted with managing a chronic disease that will persist for many decades due to lack of a cure. While there has been significant progress with the provision of life-prolonging and life enhancing combination ART to PLWHA, the number of newly infected individuals with HIV far outstrips the reduction in HIV related morbidity and mortality in Africa.^3^

Religion, and more specifically faith-based organisations (FBOs) such as churches, mosques or synagogues, can (a) have an effect on socio-cultural factors that increase or decrease the risk of infection in society; and (b) offer preventative interventions to their followers or the wider community. FBOs spend a large proportion of their time engaging with the community thereby shaping social norms, attitudes, beliefs and people's reality with regards to sexual self-understanding; making them crucial partners in HIV/AIDS prevention.^4^ FBOs often are a focal point of community life whose influence spreads beyond the traditional role of offering spiritual guidance and comfort. Therefore, in order to successfully address public health issues, FBOs are an obvious partner in preventative activities because of their influence within the African community.^5^ However, the HIV prevention methods of FBOs have been incompatible with the approach of other stakeholders. FBOs often emphasise abstinence and faithfulness as the only strategies for HIV prevention, whereas other stakeholders mainly focus on condom promotion.^6^ The latter approach continues to face opposition from FBOs, since FBOs argue that sexuality cannot be detached from the core values of love, marriage and pro-creation and that by promoting condom use these other stakeholders are encouraging sex outside marriage and promiscuity.^7^

Improved HIV knowledge, the delay of sexual debut and decreased extra- and pre-marital sex is associated with religious commitment.^8^ However, the strong condemnation of condom use potentially reduces knowledge, skills and the willingness of members to use condoms during risky sexual behaviour.^8^ Furthermore, to remain members of good standing within the church, people have to behave in a proscribed manner or face discipline, meaning that the fear of falling out of favour with the church can positively affect their risk-taking behaviours whilst at the same time inhibit the disclosure of such behaviour.^8^ This therefore increases the risk of HIV spread, because PLWHA cannot disclose their sero-status to their partners, or use condoms.^6^

## Methods

A literature search was conducted for English language articles published between 2006 and 2016 using relevant key words (Table 1).

**Table 1:** Keywords

Search term:	Affiliated terms
HIV	hiv or aids or acquired human immunodeficiency syndrome or human immunodeficiency virus or reproductive health
Faith-based organisation	faith-based organi*ation* or faith based organi*ation* or religion* or church* or mosque*
Influence	influence* or impact* or effect* or affect*
Strategies	strategy* or initiative* or intervention*
Sub-Saharan Africa	sub-Saharan africa or developing countr* or less developed countr* or “low-income countr*”

**Figure 1:**
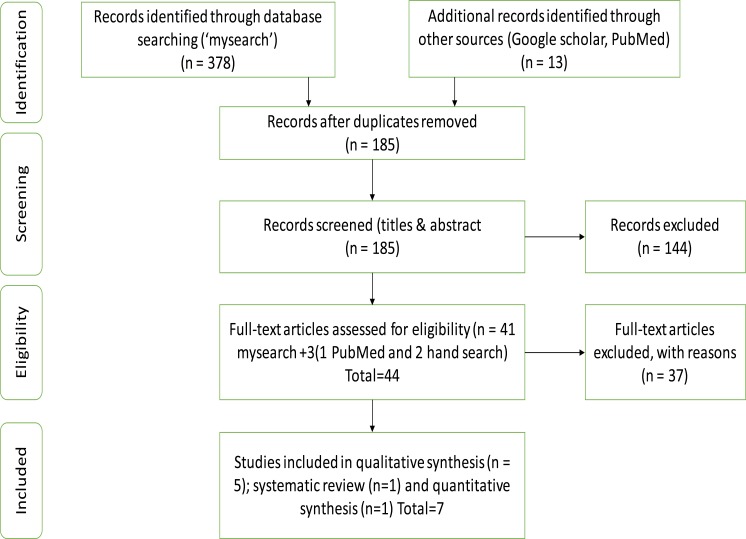
PRISMA flow diagram Key: n is number of studies included at that level

The Bournemouth University (UK) main search engine ‘mysearch’ was used as this incorporates all relevant databases (Table 2). Additionally, studies were retrieved by searches within Google scholar, PubMed and the British Library. Reference lists of included papers were also checked, and citations in key papers were hand searched^9^. The search process is presented in a PRISMA flow diagram in figure 1. EndNote software was used to store all the search results.

**Table 2:** Databases included through ‘mysearch’

Nos.	Name of Database	Hit Count
1	Social Science Citation Index	66
2	Science Citation Index	54
3	MEDLINE Complete	35
4	PsycINFO	29
5	CINAHL Complete	15
6	SocINDEX with Full Text	14
7	JSTOR Journals	8
8	Arts and Humanities Citation Index	7
9	Science Direct	7
10	Scopus	5
11	Business Source Complete	3
12	Environment Complete	3
13	British Library EthOS	3
14	Education Source	3
15	SCiELO	2
16	ERIC	1
17	Communication Abstract	1

## Study selection and data extraction

After assessing the relevance of each article separately against the inclusion criteria (Table 3), the authors came up with a list of studies with reasons for inclusion or exclusion. CASP (Critical Appraisal Skills Programme) tools were used to assess the quality of the studies.^10^ Before the critical appraisal of each paper, the CASP tools were piloted using two studies to see if they “work”. Two authors independently piloted the CASP tools. After completion of the quality assessment, responses were compared, differences discussed and issues resolved.

**Table 3:** Inclusion and exclusion criteria

Inclusion criteria	Exclusion criteria
HIV prevention strategies i.e. condom use, mother-to-child transmission, HIV testing and counselling, HIV disclosure, stigma discrimination reduction, sex education, advertising and campaigns	HIV treatment interventions i.e. ARTs and ARVs
FBO or churches or mosques or temple or any other place of worship involved in either of the prevention strategies	HIV prevention strategies that affiliated to or lead and organized by secular non-government organisations
Participants in the studies were either affected or infected with HIV and were affiliated or belonged to a religion	Participants not affected or infected with HIV and were not affiliated or belonged to any religion
Prevention strategies were being implemented in sub-Sahara Africa	Prevention strategies that were being implemented outside sub-Saharan Africa
Studies published in English between 2006 and 2016	Non-English language studies published before 2006 and those lacking publication date/authors

Table 4 summarises the data extracted from the studies. The data extraction form was also piloted on two studies by the first author. To ensure accuracy and completeness of the data extracted, the other authors cross-checked the extracted data and necessary amendments were made after discussions. Thematic analysis generated the themes that answered our research question: What is the influence of FBO's on HIV/AIDS prevention strategies in sub-Sahara Africa? Permission to conduct the systematic review was sought from Bournemouth University Ethical Committee.

**Table 4:** Summary of selected studies

Details of studies included in the review						
Nos.	Reference	Aims and/or objectives	Setting and Population	Type of HIV prevention intervention	Authors outcome and Limitations of study	Reviewers' conclusion
1	Eriksson et al. 2014^11^	Describe messages relating to sexuality and HIV prevention, given to young people by three Christian organisations and how these relate to young people's lives.	KwaZulu-Natal, South Africa Young people 15–24 years attending church conference and affiliated to e RC Church, Lutheran Church, or Assemblies of God.	Sex Education	Most perceived themselves at risk of HIV (53 %). Premarital sexual abstinence was most frequently (88 %) reported prevention message, followed by faithfulness (23 %), HIV testing (18 %) & condom use (17 %). **Limitations:** Open invitation to young people attending church conference i.e. systematic sample, hence impossible to give a response rate.	Study seems valid and reliable. The aim is clearly highlighted and seems answerable. Data collection and analysis methods are discussed in detail. Findings are also explicit and discussions are adequate.
2	Eriksson et al. 2013^12^	Gain deeper understanding of how young people perceive and reflect on messages from churches regarding premarital sex in context of their own realities	RC Church, Evangelical Lutheran Church and Assemblies of God. Durban in KwaZulu-Natal, South Africa Church-attending youth 13–20 years	Sex Education ABCs (Abstain, Be faithful and Condom use) of HIV prevention	Church messages were on abstinence thus not addressing sexually active youths. Church messages did not address contextual factors such as peer pressure, drugs & alcohol i.e. main reasons youth become sexually active. Young women were given more information on sexual issues than men. HIV testing was only considered just before marriage as it was used to detect unfaithfulness **Limitations:** Wide age range (13–20 years), attitudes on premarital sex vary depending on age.	Study seem to be rigorous, researcher has justified every decision made while conducting the study. However, researcher's relationship with participants is not clearly described.
3	Trinitapoli (2009)^13^	1. Identify overall patterns and variations in what religious leaders teach about HIV and about sexual behaviour. 2. Assess how religious organisations impact behaviour of individuals.	Malawi Religious leaders	Education ABCs (Abstain, Be faithful and Condom use) of HIV prevention	Over 88% of religious leaders report preaching about morality weekly basis;over70% report addressing sexual morality, AIDS, and illness weekly. Only 27% of religious leaders reported ever advising members to use condoms, and 95% reported privately advising members on individual basis to cease promiscuous behaviour. Condom use was low among sexually active respondents.	Aims are clearly highlighted and seem to reflect the topic of research. The selection process of participants is clear as well as the data analysis process. Overall, study seems to be rigorous as all the decisions made during the research and how they affect study have been justified hence valid.
4	Miller, A. N., and Kyalo, N. (2013)^14^	1.Investigate factors affecting denominational differences in sexual attitudes and behaviour of youths, 2. Investigate specific aspects of church environment associated with differences in response of youths to church messaging about sexual behaviour.	Church-going youths 16–21 in Nairobi, Kenya	Teachings and messages on sexual behaviour	All explained that their churches taught that sex is good, but should be saved for marriage. Condoms were viewed by their churches as promoting immorality. Some youths held different views than their churches about condom use. They agreed that youths should not have sex before marriage but condoms should be available as last resort. Youths identified curiosity, peer pressure, media, and alcohol as contributing factors to premarital sex not addressed by church. **Limitations:** Findings are not generalizable.	The problem statements agree with the title and seem to be of educational significance and the objectives were generally answerable. In addition, the methods used to gather data were clearly explained and the findings were well organized and reported objectively. Overall, every decision made during the research was justified.
5	Campbell et al. 2011^15^	Determine the role churches currently play in contributing to HIV/AIDS-related stigma.	Systematic review in Africa	Stigma reduction	HIV prevention messages often limited to abstinence/fidelity clashing with ‘mainstream’ HIV prevention campaigns. Church leaders regarded pro-condom health messages as sinful, based on their belief that those who adhered to church teachings would not need condoms. This implicitly stigmatised anyone wishing to use a condom. **Limitations** Only peer-reviewed literature, important findings identified by non-academic frontline health and welfare practitioners which are often published in non-academic ‘grey’ literature could have been missed.	The purpose of the study was clearly and concisely stated and seemed to agree with the title. The author's objectives were answerable and the search strategy clearly explained. Findings were well organized, sectioned, and reported objectively and the limitations of the study highlighted. Overall the study was rigorously done.
6	Rankin et al. 2008^16^	Describe perceived power and influence of religious groups on risk taking and HIV mitigation behaviours church members.	Blantyre urban and periurban area and in Lilongwe, capital of Malawi. National and central leaders from 5 faith-based organisations	Stigma reduction Messages on condom use ABCs (Abstain, Be faithful and Condom use) of HIV prevention	FBO leaders emphasized abstinence and marital fidelity, faithfulness as a female virtue, condoms promote sin, and demonization of government's message on condom use.	In-depth description of data analysis process. There is a clear statement of how themes were derived from data thus increasing validity of study. No justification of the research design.
7	Root (2009)^17^	Explore HIV/AIDS-related stigma and role that religiosity &church participation may play in lives of PLHIV.	Swaziland Church attenders. Most participants were accessed through an HIV-support group and a government health facility that provided HTC and low-cost ART	HIV self-disclosure in church settings	Seven participants (25%) began attending church services either while they were sick or after HIV diagnosis and 46% reported making a greater effort to attend church services post-diagnosis. 11 individuals (39%) who knew they were HIV-positive said they had disclosed to their pastors. Three of these disclosures were made to pastors' wives. **Limitations:** Findings are not generalizable. Selection bias occurred as purposive sampling was of individuals who were currently attending church. Thus those for whom becoming HIV-positive led to a cessation of church attendance were not accessed.	Aim relevant to study. Researcher has given reasons as to why chosen participants were most appropriate. The selection process is clearly described. Data collection is clearly discussed and the method made explicit. There is an adequate discussion of the results. Therefore, study seems reliable and trustworthy.

## Results

Seven studies met all inclusion criteria and were reviewed. All conducted between 2006 and 2016. Papers were excluded on the grounds of not covering sub-Sahara African countries, non-English language or lacking a prevention strategy or intervention that is faith-based. Results of the study selection are shown on the flow diagram in Figure 1.

## Description of Included Studies and Participants

All studies except two were qualitative. Three of the seven studies were conducted in Southern Africa (two in Republic of South Africa and one in Swaziland), two in South-Eastern Africa (Malawi), one in East Africa (Kenya), one was a quantitative study and one a systematic review in Africa. The participants were mainly young people in faith communities or those affiliated to a church. Religious leaders were also participants in the selected studies. Table 4 summarises the included papers.

Table 5 summarises the key themes from the included papers. However, for the purposes of focus within this paper, the themes ‘Wiser decisions regarding sexual behaviour: Condom use’ and ‘Minimal influence on multiple relationships’ were focused on.

**Table 5:** Summary of themes in selected studies

Themes	Studies/references
Wiser decisions regarding sexual behaviour: Condom use	Eriksson et al. 2014^11^; Eriksson et al. 2013^12^; Trinitapoli 2009^13^; Miller & Ngula 2013^14^; Campbell et al. 2011^15^; Rankin et al. 2008^16^
Minimal influence on multiple relationships	Eriksson et al. 2014^11^; Eriksson et al. 2013^12^
Contextual factors not addressed during teachings	Eriksson et al. 2013^12^; Miller & Ngula 2013^14^
Increase in HIV testing	Eriksson et al. 2014^11^; Eriksson et al. 2013^12^; Root 2009^17^
Abstinence and fidelity: Gender differences	Eriksson et al. 2014^11^; Eriksson et al. 2013^12^; Campbell et al. 2011^15^; Rankin et al. 2008^16^
Increase in HIV disclosure	Root 2009^17^

## Wiser decisions regarding sexual behaviour: Condom use.

Wiser decisions on sexual behaviour was identified as an influence of the church teachings on HIV prevention among church-attenders as they paid more attention when making decisions regarding their sexual behaviour. Condom use was the most discussed sexual behaviour and contradicting views were clearly evident. Condom use was controversial as religious leaders held different views. Some religious leaders disapproved of condom use while others cautiously approved condom use to their church members in private.

Religious leaders who disapproved condom use strongly shared the views of many lay Africans, where they dismissed condom use as an impractical HIV prevention strategy that reduces sexual pleasure.^13^ Others had a tendency to equate condom use to promote infidelity and promiscuity thus talked about condoms as promoters of sin during their church teachings. Furthermore, their religious teachings link HIV, condom use and immorality hence suggesting that only non-believers are at risk of HIV infection. This therefore leads to church-going youth perceiving themselves to have little or no risk, leading to inconsistent protective practices.

Religious leaders who are not in complete disapproval of condom use gave advice cautiously to church members. This was seen in religions where they hold a strong negative policy on condom use hence the need to privately advise members of their congregation on condom use. Trinitapoli^13^ argues that young adults who attend religious congregations where their leaders privately advise on condom use are more likely to abstain.

However, young people in faith communities often held different views from their churches on condom use. Although they agreed that youths should not have sex before marriage, they considered the use of condoms as a last resort. It could therefore be suggested that youths engage in less risky behaviour when it comes to HIV prevention. As much as their churches discourage on condom use, they prefered to go against the teachings and practise safer sexual behaviour thus decreasing their risk of HIV infection.

## Minimal influence on multiple relationships

Another theme which emerged from the papers was the influence of the church teachings on multiple relationships, especially among men. Eriksson and colleagues^11^, ^12^ found that more males than females reported either being in a relationship or having had a relationship. Additionally, both male and female respondents agreed that it was more acceptable for men to have multiple sexual partners as illustrated below:

“…honestly speaking, the truth is, it's hard to abstain” (Boy, AOG: 2). Both young men and women agreed it was more acceptable for men to have multiple sexual partners” (12: -, p 461).

This indicates that gender differences in the number of partners among the religious youth may be similar to those found in youth in the wider society suggesting that affiliation to a religion had a minimal effect on multiple relationships among faith communities.

## Discussion

### Wiser decisions regarding sexual behaviour: Condom use

Due to the church teachings and messages on sexual behaviour, church-going youth paid more attention when making sexual behaviour decisions.^11^ Similarly, in a peerled education on sexual behaviour among Malaysian university students aged 18–24 years, participants were found to have sound knowledge and were confident in making wiser decisions regarding sexual behaviour after the intervention.^18^ Therefore, it could be concluded that education interventions on HIV prevention and sexual behaviour among the youth has a strong effect on their sexual behaviour and decisions.

Condom use was the most discussed sexual behaviour and contradicting views was clearly evident. There were those religious leaders who completely disapproved of condom use whereas others cautiously advised members on using condoms. Although young people attending church regularly agreed that sex outside marriage is not acceptable, they still considered condom use as a last resort. Campbell et al. found that youths who self-identified as being religious were less likely to use condoms compared to those who were less attached to a religion.^15^

Therefore, to compare the use of condoms among church-going youths and other youths in general, university students were the comparative group because universities are a priority target area for promoting and enhancing healthy sexual behaviour amongst youths. Additionally, university students are youths who are impressionable, may not have adequate or consistent income, and are living without consistent adult supervision for the first time in their lives. These influencing factors com pound with peer pressure make them highly vulnerable to acquiring HIV. Students in the Democratic Republic of Congo aged 18–33 years were less likely to use condoms consistently despite having awareness and access to them. This does not usually translate to the practice of consistent condom use possibly because one of the barriers to condom use amongst students was the influence of their religious advice to not use them. Religion is a major influence in the sexual behaviour of youths.^19^ Furthermore, trust in partners was also a barrier to condom use raising issues of a lack of trust or uncertainty within a relationship making it more difficult for partners to discuss condom use. However, studies in Africa have found that the level of religiosity is a significant predictor of good attitude towards condom use, abstinence and the knowledge towards HIV prevention.^20^,^21^,^22^

The low and inconsistent use of condoms amongst the African youth is a crucial factor involved in the large proportion of all new HIV infections occurring in the under 25 year olds.^23^ It is therefore necessary to identify the determinants of risk and protective interventions that are appropriate to this age group. Additionally, the need for combined HIV prevention strategies and interventions that address these specific factors amongst youths, would be the best approach for reducing the prevalence of HIV in the under 25 year olds.

### Minimal influence on multiple relationships

It was clear that despite the religious teachings on HIV and sexual relationships among young people, it was more acceptable for men to have multiple sexual partners than it was for women. This could be because the consequences of pre-marital sex are different for young men and women.^11^,^12^ Men were regarded as “heroes” among peers for having multiple sexual relationships. In contrast, women who were sexually active were regarded as weak and had a bad reputation.^12^

In Africa, cultural beliefs enforce the view that it is acceptable for a man to have more than one sexual partner. The acceptable norm of men to have multiple relationships is attributed to African societal norms where infidelity is generally accepted for men but not for women.^24^ In addition, the acceptable norm of men to have multiple sexual partners is also attributed to the authority men have over their wives. This authority is established through bride wealth payments and is reinforced by numerous other means, such as the reliance of wives upon their husbands for economic and material support.^25^ All studies suggested that it is acceptable for men to have multiple sexual relationships. However, the sexual behaviour among the participants in Bingenheimer's study^25^ was self-reported therefore some reporting bias was likely. Some suggested that affiliation to a religion had minimal influence on multiple relationships among faith communities as gender differences in the number of partners among religious youth is similar to those found in youth in the wider society.^11^,^12^, ^21^ Therefore, men need more awareness on the risks of having multiple sexual partners given that their behaviour is culturally bound.

## Conclusion

In sub-Saharan Africa, governments often have extremely limited resources to reach all communities in terms of HIV prevention and treatment. Given the accessibility of local FBOs and the coverage of religion among the population, FBOs are potentially important players in HIV prevention and are often in the forefront of responding to the HIV/AIDS epidemic. FBOs can help deliver health promotion messages and materials that are affordable and acceptable. By exploring the influence FBOs have on HIV/AIDS prevention strategies, this research has contributed to a greater understanding of the overall faith-based response to HIV/AIDS in sub-Saharan Africa and how they shape the sexual behaviour of young people. It is hoped that by doing so, FBOs will be better at identifying how best to educate their congregations on HIV prevention thereby improving the overall goal in the fight against HIV/AIDS.

HIV prevention messages are still in line with the already existing traditional messages regarding sexuality. Therefore, FBOs should incorporate amore comprehensive sexual education that addresses the social contexts that makes church-going youth and youth in general vulnerable to HIV infection. The educational messages and teachings seem inappropriate as youths are still sexually active. Hence FBOs need to strengthen their capacity to educate young people in a more holistic way about sexuality and HIV prevention. Furthermore, FBOs should also consider educating and creating awareness on the risks of multiple relationships particularly to those men whose risky behaviour is culturally bound.

More resources should be given to support FBOs in their strategies to prevent HIV infection among faith communities thus improving services. Additionally, governments should offer training to religious leaders on how to pass on their teachings and messages about HIV prevention strategies. This may make them improve their prevention strategies and enhance the understanding of the role FBOs play in HIV/AIDS prevention, care and support. Although our aim was to conduct a review with good quality studies that would be valid and generalizable to sub-Saharan Africa, we acknowledge that this review is limited by the possibility of having missed relevant studies within the timescale and resources available.
